# Decentralized but Globally Coordinated Biodiversity Data

**DOI:** 10.3389/fdata.2020.519133

**Published:** 2020-10-23

**Authors:** Beckett W. Sterner, Edward E. Gilbert, Nico M. Franz

**Affiliations:** School of Life Sciences, Arizona State University, Tempe, AZ, United States

**Keywords:** data aggregation, ontology alignment, biodiversity data, communities of practice, data intelligence, decentralization, knowledge commons, systematic biology

## Abstract

Centralized biodiversity data aggregation is too often failing societal needs due to pervasive and systemic data quality deficiencies. We argue for a novel approach that embodies the spirit of the Web (“small pieces loosely joined”) through the decentralized coordination of data across scientific languages and communities. The upfront cost of decentralization can be offset by the long-term benefit of achieving sustained expert engagement, higher-quality data products, and ultimately more societal impact for biodiversity data. Our decentralized approach encourages the emergence and evolution of multiple self-identifying communities of practice that are regionally, taxonomically, or institutionally localized. Each community is empowered to control the social and informational design and versioning of their local data infrastructures and signals. With no single aggregator to exert centralized control over biodiversity data, decentralization generates loosely connected networks of mid-level aggregators. Global coordination is nevertheless feasible through automatable data sharing agreements that enable efficient propagation and translation of biodiversity data across communities. The decentralized model also poses novel integration challenges, among which the explicit and continuous articulation of conflicting systematic classifications and phylogenies remain the most challenging. We discuss the development of available solutions, challenges, and outline next steps: the global effort of coordination should focus on developing shared languages for data signal translation, as opposed to homogenizing the data signal itself.

## Introduction

After several decades of efforts toward a centralized and unified global biodiversity data infrastructure, the broader community is now in a reflective moment about where to go next and what it can learn from past successes and failures. Inspired by big science efforts like the Human Genome Project, major biodiversity initiatives have set the goal of aggregating all data about where and when different biological entities—most typically “species” in our context—are located, in order to provide critical insight into global problems such as rapid biodiversity loss and climate change (Peterson et al., 2010; Devictor and Bensaude-Vincent, 2016; IPBES, 2019; Wagner, 2020). However, there are an exceptionally large and heterogeneous set of stakeholders for this emerging biodiversity knowledge commons (Adams et al., 2002; Strandburg et al., 2017), making effective governance a critical, ongoing challenge (Alphandéry and Fortier, 2010, Turnhout et al., 2014). The present moment marks a pivotal opportunity to examine how a new, *decentralized* approach may better provide the “flexibility both to accommodate and to benefit from this diversity [of contributors], rather than seeking to implement a prescriptive programme of planned deliverables” (Hobern et al., 2019, p. 9)—as recommended by a recent report from the second Global Biodiversity Informatics Conference.

Next-generation solutions, we argue, must proactively reckon with the limitations of overriding local differences in the production and meaningfulness of biodiversity data (Sterner and Franz, 2017; Franz and Sterner, 2018; see also Gallagher et al., 2020). Not only is the fundamental nature of biodiversity highly contested, our knowledge of many taxonomic groups is vastly incomplete, changing at an unprecedented rate, and upending historically established methodologies (Hinchliff et al., 2015; Hortal et al., 2015; Whitman, 2015; Peterson and Soberón, 2017). As a result, the basic categories biodiversity scientists use to communicate, integrate, and reason about their data are frequently disputed or unstable (e.g., Vaidya et al., 2018). More broadly, biodiversity loss is also a good example of a “wicked” problem: “one cannot understand the problem without knowing about its context; one cannot meaningfully search for information without the orientation of a solution concept; one cannot first understand, then solve” (Rittel and Webber, 1973, p. 162). Incorporating the possibility of dissent and local customization into the fundamental architecture of the data ecosystem may therefore prove crucial to harnessing the data revolution to address biodiversity loss (Sterner et al., 2020).

The historically dominant paradigm, by contrast, has depended upon a stable or coherently evolving consensus among multiple scientific communities about the best way to classify natural phenomena (Godfray, 2002; Smith et al., 2007; de Jong et al., 2015; Ruggiero et al., 2015; Sterner et al., 2020). Following this best-consensus model, first-generation big biodiversity data projects have generally prioritized scale and comprehensiveness, launching large initiatives to centralize access through national or global web portals (Wieczorek et al., 2012). However, these efforts have struggled to address widespread deficiencies in data quality, in part because they have purchased global data aggregation at the price of imposing homogeneity on data that were generated using different standards and classification schemes (Mesibov, 2013, 2018; see also references in Franz and Sterner, 2018). Responsibility for persistent dissatisfaction with the quality of aggregated biodiversity data cannot be simply or even primarily assigned to the globally distributed set of organizations maintaining such data collections: many deficiencies occur *through* the aggregation process, i.e., at higher levels in the data flow trajectory. The centralized governance approach moreover provides limited mechanisms for experts to advance curation and innovation addressing fitness-for-use requirements for diverse stakeholders (Franz and Sterner, 2018).

Given the many diverse projects and data sources involved in the biodiversity data commons, some sort of regional to global coordination is nonetheless clearly necessary (Turnhout and Boonman-Berson, 2011). The primary unit of biodiversity data being aggregated across these contexts is an observation event of an organism at a particular place and time, vouchered with a preserved material specimen, photograph, or in some cases DNA sequence. Historically, the dominant source for such “occurrence data” —as named by the widely adopted Darwin Core standard (Wieczorek et al., 2012)—have been natural history collections and ecological surveys hosted and carried out around the world. The past several decades have introduced major new data sources, including enthusiast science efforts such as eBird or iNaturalist, remote sensing technologies such as camera traps or satellite imaging, and automated genetic sequencing efforts such as DNA barcoding and environmental metagenomics.

**Figure 1:**
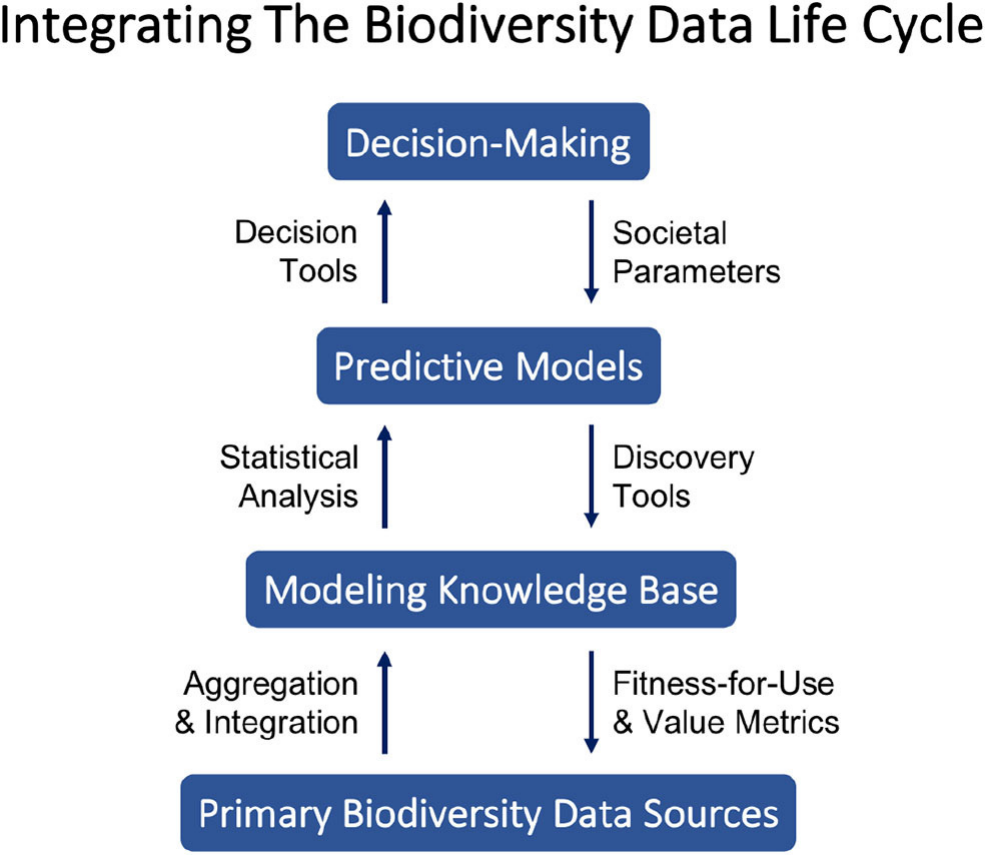
Each level (blue rectangles) represents a broad class of products or outcomes exchanged among researchers, decision-makers, and society members. The arrows linking levels describe information processes important to generating value across levels.

In response to these complexities, we present a novel philosophy of decentralized coordination for biodiversity data science integrating recent advances in the field. Our approach embodies the spirit of the Web as “small pieces loosely joined” (Weinberger, 2008) and we argue for its broader potential to strengthen integration across the biodiversity data life cycle. Reflecting the societal importance and urgency of continued biodiversity loss, we characterize the biodiversity data life cycle here in terms of a positive feedback loop connecting primary data sources and decision-making. Figure 1 illustrates key components and interactions we identify as central to integrating the biodiversity data life cycle from primary data collection to decision-making. The figure can be interpreted as a schematic diagram to be filled in with particulars for a given situation, e.g., with the design of a new protected area as the decision to be made, models for species richness and spatial niches as the predictive models, and GIS data layers as the modeling knowledge base.

Rather than building a top-heavy global infrastructure with weak connections to existing institutions and efforts at local and regional scales, we argue for innovating and promoting competing designs to empower and coordinate both existing and newly emerging communities of practice. Biodiversity data infrastructure and informatics tools should then reflect the forms of social engagement, imagination, and alignment within and among communities of practice that best promote biodiversity learning and high-quality data (Wenger, 1998, 2000). By necessity, each community will have a taxonomic, regional, or institutional reach below that of a single, global network but will also have the capacity to grow or shrink dynamically to reflect changing resources and needs. Cross-community coordination toward a more global reach can happen simultaneously through the articulation of classification alignments enabling translation of biodiversity data across evolving or conflicting, locally maintained perspectives. Decentralized coordination therefore depends on the relatively lossless translation of data but not on a stable, shared theory of the world or pool of settled knowledge.

Our core aims in this paper are thus to: (1) critically review how centralizing control over the data aggregation process and outputs limits opportunities and incentives for continuous growth and fitness-for-use; (2) synthesize recent advances to articulate a positive alternative model based on facilitating lateral data sharing among a distributed network of portals. We start in Section Growing the Biodiversity Data Commons by drawing a distinction between centralized and decentralized governance approaches, which we then apply in Section Reframing the Critique of Globally Centralized Biodiversity Data to synthesize and extend previous critiques of the centralized approach. Sections A Model for Decentralized But Globally Coordinated Data Aggregation and Developing Socio-Technical Infrastructure to Implement Coordination pick up the paper's second aim by raising some key challenges for maintaining coordination under a decentralized approach and articulating a path forward based on existing or in-development tools and methods.

## Growing the Biodiversity Data Commons

The design of biodiversity data infrastructure has ramifications beyond the properties of any single aggregated dataset it produces or hosts. To this point, this section introduces a distinction between centralized and decentralized strategies for global biodiversity data aggregation. Since decentralized approaches have received less attention in this context, we also highlight the important role that communities of practice play in the success of knowledge commons. This conceptual and empirical background will provide the basis for evaluating and designing better systems for biodiversity knowledge commons in the following sections.

The Global Biodiversity Information Facility (GBIF) now processes and serves over 1.5 billion occurrence data points for users to search and download, aggregated from a wide range of citizen science projects, museum collections, and other organizations. Similar large national or continental aggregators exist, such as Integrated Digitized Biocollections (iDigBio; Hanken, 2013) and DataONE (Michener et al., 2012), as well as for specific taxa such as the Avian Knowledge Network (Iliff et al., 2009). Aggregation and curation at this level relies heavily on broad adherence to the Darwin Core standard for publishing occurrence data, which imposes minimal metadata requirements and categories (Wieczorek et al., 2012). Relying on this minimal consensus standard, aggregator portals can then provide centralized access points for datasets sourced from a wide range of repositories, collections, and databases.

However, simply adding more data to a problem—e.g., by aggregating observations across many sources—unfortunately does not necessarily lead to improved predictions or discoveries, despite claims that suggest theoretically informed statistical analyses and experimental designs are no longer relevant (Philippe et al., 2011; Lazer et al., 2014; Leonelli, 2016; Sterner and Franz, 2017). “The real source of innovation in current biology,” argues philosopher of science Sabina Leonelli, “is the attention paid to data handling and dissemination practices and the ways in which such practices mirror economic and political modes of interaction and decision making, rather than the emergence of big data and associated methods per se” (Leonelli, 2016, p. 1).

For example, consider how new norms around open data become institutionalized in science. Proactive individual adoption of open data practices matters for the success of large cyberinfrastructure projects, such as Dryad (White et al., 2008) or DataONE. From a top-down policy perspective, compliance with open data principles is an obligation for individual scientists as part of their funded research. Addressing these concerns, survey studies addressing biodiversity data sharing have primarily focused on scientists' individual attitudes or behaviors (e.g., Enke et al., 2012; Schmidt et al., 2016). From a bottom-up community perspective, however, following norms about data use and sharing is one aspect of participating in the community's broader scientific identity, culture, and social organization (Wenger, 1998; Aronova et al., 2010; Strasser, 2011). Research has shown these community-level variables have a strong impact on the success or failure in the related domain of collaborative open source software projects (Schweik and English, 2012). Some of the most successful community curation projects for genome data have also emerged as bottom-up collaborations that use wiki technology to layer information on top of centralized sequence repositories (Lee, 2017). This example illustrates the value of looking beyond abstract principles or policies to examine how situated rules and practices affect the ways actors engage with each other and shared resources.

The literature on knowledge commons provides a useful framework and body of empirical studies for this purpose. The idea of a “commons” refers to an institutionalized arrangement for the use and management of a shared resource among multiple actors (Strandburg et al., 2017). A commons therefore denotes “a form of community management” for a resource; not the resources, community, or place being managed (Strandburg et al., 2017, p. 10). While the term is also often used in a loose metaphorical way, several types of commons have been subject to extensive theoretical and empirical research, including natural resource commons and knowledge commons (Ostrom, 2010; Schweik and English, 2012; Strandburg et al., 2017). A knowledge commons in particular is defined as “the institutionalized community governance of the sharing and, in many cases, creation of information, science, knowledge, data, and other types of intellectual and cultural resources” (Strandburg et al., 2017, p. 10).

As we zoom in to look at how scientific data are created, shared, and curated in practice, it is clear that the governance of knowledge commons operates in multiple dimensions simultaneously and may reflect heterogeneous strategies for different aspects of the data and modes of engagement. One reason is that actors can participate in the commons in multiple ways, including adding, altering, removing, restricting, using, and exchanging access rights to the shared resources (Frischmann et al., 2014). Another reason is that the data themselves are complex objects whose properties and scientific significance often vary with context (Franz and Thau, 2010; Millerand et al., 2013; Leonelli, 2016; Lee, 2017). Metadata are key in order to find and use scientific data online, but the language scientists use to describe the observations they have made are often highly local and dependent on tacit knowledge (Leonelli, 2016; Sterner and Franz, 2017).

An important distinction among knowledge commons is whether authority over the pooled resource is allocated in a centralized or decentralized way. Where totally centralized governance would put all decisions regarding actors' access, use, and contribution rights in the hands of a single organization or group, totally decentralized governance would instead give full autonomy in each of these respects to local organizations or communities (Brown and Grant, 2005). Computer ontologies in the life sciences, for example, are typically developed and maintained under centralized governance by a single consortium in charge of formulating and maintaining consensus definitions for the associated terminology (Smith et al., 2007; Wieczorek et al., 2012; Millerand et al., 2013). As we use the term here, a computer ontology provides standardized metadata vocabularies organized into formal logical relationships that allow users to categorize and reason about data in a machine-readable format. Many of the largest bio-ontologies, such as the Gene Ontology, are members of the Open Biomedical Ontologies (OBO) Foundry, which historically has been associated with a metaphysically realist approach to selecting and defining metadata vocabularies (Smith et al., 2007; Sterner et al., 2020).

A range of intermediates also exist between extremes of centralized and decentralized approaches, reflecting different forms of governance arrangements and the multiple aspects of actors' relationships to the knowledge commons (Fisher and Fortmann, 2010; Ostrom, 2010; Contreras and Reichman, 2015). The collaborative software development and repository service GitHub, for example, provides an intermediate option for programmers where updating and releasing a software project is controlled by a single individual or group, but any individual is able to initiate a new copy (“fork”) of the code and edit it in a decentralized fashion without any obligation to re-integrate the code updates back into the main project.

While highly centralized control of the data aggregation process and its outputs may appear to be necessary for building successful shared infrastructure at such large scales, this is not borne out empirically (Hess and Ostrom, 2006, 2007; Frischmann et al., 2014; Strandburg et al., 2017). In addition, contrary to conclusions suggested by early game theory models, centralizing control over a shared resource pool is neither necessary nor the most common way to prevent overexploitation leading to collapse (Wilson et al., 2013). We will also argue in the next section that centralization can *undercut* the ability of individuals and local communities to function and contribute valuable biodiversity information, expertise, and time.

Furthermore, despite the apparent potential for exploitation or anarchy, decentralized governance can succeed when robust local institutions exist that teach, enforce, and maintain rules (either formal or informal) about appropriate actions. The concept of a “community of practice” provides a useful way of understanding how people negotiate these rules and their application over time (Wenger, 1998, 2000). A community of practice provides the interpersonal and institutional scaffolding needed for people to be recognized, trained, and leaders in a particular skill set. The possible subject matter for a community of practice ranges broadly; including e.g., claims processing in insurance, macro photography, and college teaching. In biodiversity science, examples would include researchers working to advance the classification of a taxonomic group such as birds, coordinating on the conservation of a particular ecosystem, or working to improve informatics tools for sharing occurrence datasets. The relevant community is restricted, though, to a particular group of people (not necessarily in one geographic place) who interact regularly as part of learning, applying, and improving these skill sets. The community is therefore connected by the practice as a shared matter of concern, such that they are involved in a joint enterprise based on mutual interactions and a shared repertoire. It forms a group to which people can belong through their engagement and alignment in joint projects using the relevant skill set and through a shared vision for the community's aims and identity.

## Reframing the Critique of Globally Centralized Biodiversity Data

We now turn to describe key limitations of the dominant, centralized governance for global biodiversity knowledge commons. The primary functionality provided by current biodiversity aggregators is arguably serving as centralized portals for finding and downloading occurrence datasets (Hobern et al., 2019). This is clearly a desirable feature for users exploring the data available for projects outside existing thematically focused and curated collections. However, implementing this query functionality drives further choices between a centralized vs. decentralized strategy for handling data aggregation. We critique two such choices: centralized control over the metadata categories used to aggregate data, especially taxonomic classifications, and centralized control over the flow of information reflecting new curation or collections work.

One major and persistent problem with globally centralized biodiversity data aggregation is that it can significantly distort the underlying signal in heterogeneous datasets, leading to a “synthesis that nobody believes in” (Franz and Sterner, 2018). This will occur whenever occurrence records from multiple low-level sources are annotated with taxonomic name usages that are coherently applied *within* one source, but are nevertheless non-congruent *across* sources. As an example, we have shown how multiple locally and simultaneously applied meanings of taxonomic names for endangered Southeastern United States orchids in the *Cleistes/Cleistesiopsis* complex will produce misleading ecological and conservation inferences if aggregated under one centralized classification (Franz et al., 2016b; see also Peterson and Navarro Sigüenza, 1999). These issues with conflicting taxonomic labels is just one example in a broader debate about the desirability of consensus on a single classification system for a domain of data and appropriate governance strategies for managing advances and disagreements about metadata categories, especially when their definitions are justified by empirical hypotheses and evidence (Sterner et al., 2020).

**Figure 2:**
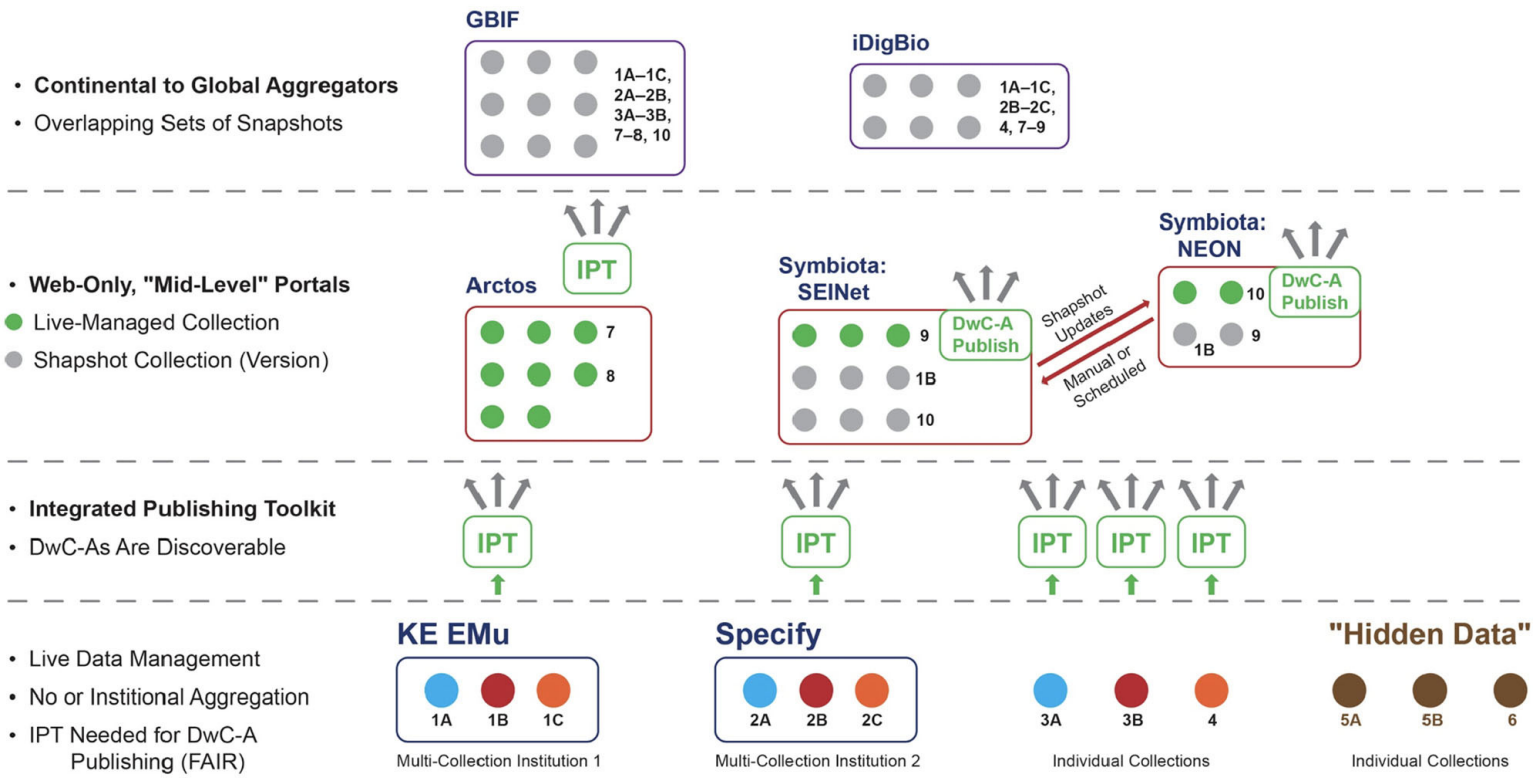
Schematic representation of biodiversity data aggregation under a centralized system. Continental to global aggregators are at the highest level, then web-only portals at the middle level, and institutional or individual collections at the lowest level. Collections pertaining to the same academic institution carry the same number with succeeding letters (1A, 1B, etc.). Abbreviations or proper names used: DwC-A, Darwin Core Archive (standard for packaging and publishing biodiversity data using Darwin Core terms); DwC-A Publish, custom DwC-A Publisher embedded in Symbiota software and portals; FAIR principles, findability, accessibility, interoperability, and reusability; GBIF, Global Biodiversity Information Facility; iDigBio, Integrated Digitized Biocollections; IPT, Integrated Publishing Toolkit (open source software tool used to publish and share biodiversity datasets through the GBIF network); Arctos, KE EMu, Specify, and Symbiota, specifically, functionally overlapping software packages for managing collections-based biodiversity data; SEINet (originally: Southwestern Environmental Information Network) and NEON (National Ecological Observatory Network), realized instances (communities of practice) of Symbiota biodiversity data portals. See text for additional explanation.

**Figure 3:**
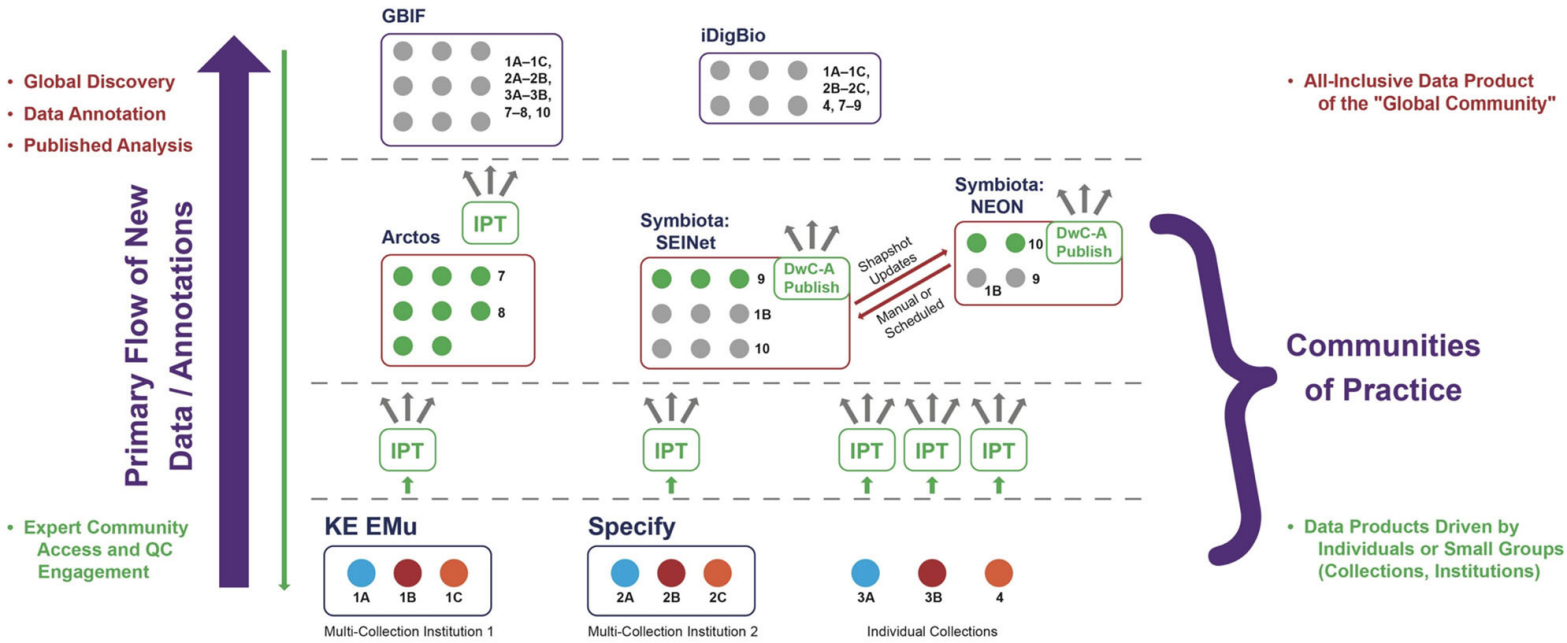
Primary flow or directionality of new data items (occurrence records) and annotations under the centralized aggregation paradigm. Whereas, much of the global data discovery and further analysis requires interaction at the highest level (left region), engagement with communities of practice is largely constrained to the low- and mid-level environments. QC, Quality Control. See text for additional explanation.

The distortion in signal created by centralized aggregation also has a social impact: it generates an unbalanced allocation of power among actors or organizations seeking to represent and propagate alternative data signals based on conflicting hypotheses or approaches in the community. To illustrate this point, we need to describe the current organization of the biodiversity data ecosystem in more detail, focusing on how data are structured in the aggregation process. Figures 2, 3 provide related, schematic representations of how biodiversity data are aggregated under a centralization paradigm. It is sufficient for our purposes to show 2–3 distinct aggregation layers, although in practice there are often additional layers and more complex data flow relationships. We mention specific institutional entities, software platforms, and information communities to illustrate broader phenomena in a concrete way (and consistent with the authors' primary expertise in North American projects).

### Low Level

At the lower levels of the aggregation hierarchy, making data packages globally discoverable according to open science standards (e.g., Wilkinson et al., 2016) requires at minimum: (1) the locally stored data records are accurately and consistently mapped to a metadata standard—in our case Darwin Core Archive format (DwC–A); and (2), packaged data records are exposed to the web via a DwC–A interface such as the Integrated Publishing Toolkit (IPT; Robertson et al., 2014). Only data package publication with the IPT or similar interfaces allows global data discovery facilitated by an Application Programming Interface (API) and further protocol-driven aggregation.

At this level, we find individual private or institutionalized collections that may or may not be digitized in some form, but in either case don't meet the criteria above. We may term these “hidden data” (Figures 2: 5A, 5B, 6).

We also find other collections that are standards compatible and accessible online through custom publishing solutions (Figures 2: 3A, 3B, 4) or software applications designed to serve multi-collections institutions such as museums, academic institutions, and environmental organizations (e.g., the KE Emu and Specify software applications; Figure 2: 1A−1C, 2A−2C). These data providers will typically *not* host occurrence records in their managed systems when the corresponding specimens or vouchers are owned by external entities.

### Mid-level

As defined here, a mid-level aggregator is explicitly scoped in its design and mission to aggregate biodiversity across only certain taxonomic groups or geographic regions below the continental scale. Mid-level aggregators also have the ability to host data from collections pertaining to multi-institutional communities of practice in the sense of Wenger (1998, 2000). Such community-driven data platforms are often referred to as *portals*. Reciprocal exchange among portals with different origins, emphases, management structures, and target communities embodies a lateral rather than hierarchical network at the middle level.

Data packages are currently managed in mid-level portals in the following two ways: (1) “live-managed,” which means that the entity owning the physical collection of specimens or vouchers has comprehensive rights *within* the pertinent portal to create new occurrence records and annotations (referred to as within-portal rights); and (2) “snapshots,” which are time-stamped *versions* of a live-managed collection exported to one or more *outside* portals where occurrence records may be annotated further, typically by actors that are not members of the physical collection-owning entity (referred to as outside-portal rights)^1^. Snapshot collections can be periodically or even automatically updated from the live-managed portal. Conversely, annotations made on snapshot occurrence records can be integrated with the corresponding live-managed collection under proper social and technical conditions.

Depending on its history and sustainability model, a portal may create and maintain its own, one-off software application or on a software platform serving multiple portals with installations hosting different data collections. Certain mid-level aggregators, such as Arctos (*https://arctosdb.org/*), may only support live-managed collections (Figure 2: 7, 8). However, the Symbiota software platform (Gries et al., 2014) supports live-managed as well as snapshot collections in the same portal (Figure 2: 1B, 9, 10) and has a built-in DwC–A publishing module.

Both of these design features of the Symbiota platform are essential for exchanging occurrence records and annotations *laterally* across mid-level portals. For example, the SEINet portal, which focuses on vascular plants in the Southwestern United States, can reciprocally exchange occurrence records and annotations with the National Ecological Observatory Network (NEON) portal, which includes information about a variety of taxa sampled across the U.S. and is intended to facilitate long-term macrosystems ecological monitoring and forecasting.

### High Level

High-level aggregators aim for both comprehensive taxonomic and geographic coverage on national, continental, or global scales; for instance all natural history collections occurrence records of North American institutions (Figure 2: iDigBio), or even all organismal records, globally (Figure 2: GBIF—Global Biodiversity Information Facility, 2019). By design and aspiration, high-level aggregators aim for aggregated occurrence data services and user communities who expect a “one-stop shop” in order to get data signals for “everything there is.”

### Implications

On the surface, the comprehensive scope of high-level aggregators appears to give them a distinct advantage over mid- and low-level providers. Moreover, high-level aggregators typically prefer to receive occurrence data packages and periodical updates thereof directly from the lowest-level instance of a data collection enabled with DwC–A publishing. This means that some collections may not be discoverable at the middle level when the collection has no data publishing arrangement with a mid-level portal.

However, high-level aggregation also frequently incurs the disadvantage of only providing collection snapshots. Whereas, globally comprehensive data discovery is favored at the high level, new data annotations are not. Instead, users sourcing their data from high-level aggregators typically download them into external web or local environments to carry out data cleaning, annotation, analysis, and publication. Because of the hierarchical aggregation design, it is exceedingly hard to “push” these value-added data annotations back down to the live-managed collections. These value-added actions tend to occur in external systems, not the software platform hosting or serving the occurrence data, and DwC–A transmission channels from source to user are generally configured to function unidirectionally. As a result, the vast majority of new annotations only flow one way, i.e., upwards, leading to a strongly *unidirectional* as opposed to reciprocal or cyclical quality enhancement system (Figure 2, left region). This unidirectional flow leads to potentially infinite, variously differing and outdated versions of “the same occurrence records” stored in unconnected systems, without adequate mechanisms to ensure annotation provenance and translation of syntactic and semantic changes.

A second disadvantage for high-level aggregators arises out of their increased separation from most biodiversity expert communities of practice, which simply do not work on all groups and at the global level. Instead, the experts and communities tend to have both taxonomic and geographic (or even political) boundaries that are much more accurately represented at low and middle levels of aggregation (Figure 2, right region). Conversely, research communities that *are* interested in analyzing all organisms at the global level tend to lack the highly contextualized expertise needed, for example, to reconcile non-congruent classification schemes inherent in biodiversity data packages aggregated from many localized sources and distinct communities of practice.

This gap in expertise may help explain why in practice centralized aggregation of occurrence records and centralized management of data-structuring biological classifications often go hand and hand.^2^ For instance, GBIF—currently the most globally comprehensive aggregator of all—is actively promoting its “backbone” taxonomy (de Jong et al., 2015) to which all aggregated data are preferentially matched (Franz and Sterner, 2018), often with considerable loss of syntactic preference and semantic context for new or contested nomenclature. GBIF thereby preferentially responds to the needs of those users who are interested in large-scale signals yet who are also likely unprepared or unwilling to add value to data packages live-managed at much lower levels.

## A Model for Decentralized but Globally Coordinated Data Aggregation

Decentralized governance for data aggregation offers an important alternative (Contreras and Reichman, 2015). While potentially better able to accommodate local communities of practice, a decentralized approach of course risks continuing the current fragmented state of the biodiversity data ecosystem. Unlike some of the most familiar examples of commons, such as aquifers or forests, knowledge commons cannot rely on natural processes to aggregate resources into a single, shared resource. Instead, aggregation must be engineered and re-engineered over time to ensure continued coordination of new data contributions and modifications to existing datasets. This sets up a critical challenge for decentralized approaches to data aggregation: how to engineer the capacity for competing hypotheses and distributed curation work without losing the connectivity required for global data sharing?

In this section, we introduce a novel conceptual model for achieving decentralized but globally coordinated data commons. Crucially, a decentralized approach permits multiple metadata systems to coexist when important to the functioning of relevant communities of practice while ensuring sufficient resources exist to allow efficient and accurate translation across locally variable metadata systems. Decentralization therefore relies on a second-order consensus about how to coordinate across local variation among communities of practice rather than a first-order consensus about a single global best option for all such communities (Sterner et al., 2020). Our conceptual model therefore extends a core tenet of philosophical pluralism into the context of knowledge commons: “What is crucial for your ability to communicate with me–to convey to me information about your beliefs, plans, or values–is not that we have a commonality of beliefs or ideas, and so stand in a consensus of some sort… It pivots on the recipient's capacity to *interpret*–to make good inferential sense of the meanings that the declarer is able to send. In the final analysis the matter is not one of an *agreement* between parties but of a *co-ordination* between them” (Rescher, 2000, p. 148, emphasis in original).

In particular, we propose that core operations of the Git model for decentralized version control provides a powerful and successful foundation (Loeliger and McCullough, 2012). Perhaps best known through its implementation by GitHub, the core Git operations allow a group of software developers to create parallel copies (“forking”) of a shared reference standard (the “master”) and edit these copies locally before merging the edits with the reference standard (via a “pull request”), which may itself have changed in the meantime. Similarly, local copies can be updated with changes from the reference standard (a “push”) by reconciling edits to the local and reference copies. Adopting these operations for a project comes with governance decisions about who has the ability to create local copies, request and approve changes to the reference standard, and push updates from the standard to local copies. Contributors to a collaborative project will generally form a community of practice, and the appropriate governance strategy within a community can vary from highly centralized to highly decentralized; indeed, communities often evolve over time as they grow or change identity (Shaikh and Henfridsson, 2017).

There are a couple key limitations, however, which need to be addressed for achieving decentralized but globally coordinated biodiversity data. One critical limitation of current Git implementations is that while they can track line edits to documents, they do not evaluate the semantic implications of those edits. In the context of software development, this means that updating the reference standard with changes in a local copy does not take into account any consequences to the program's input-output behaviors. There are heuristics to flag changes that may create problems, e.g., parallel changes across copies in the same lines of code, but these flags are based on proximity in the document, not effects on run-time behavior. In the context of data aggregation, this means that we could tell when the taxonomic authority for a specimen's nomenclatural identification has been changed but not necessarily whether this involves a change in the associated meaning of the taxon name. It is therefore essential to extend current implementations to incorporate semantically-aware conflict detection and reconciliation between datasets with different metadata classification systems. Current state of the art relies on a supervised approach to identify and handle cases where users have made changes to parallel copies of data records (e.g., Arndt et al., 2019).

A second limitation is that semantically-aware data reconciliation needs to be possible across multiple reference standards rather than solely with respect to local copies of a single reference standard. A setting where systematists maintain multiple, partially conflicting species checklists (e.g., as has been the case with birds for many decades; Vaidya et al., 2018) is most analogous to handling decentralized versioning across multiple Git projects (i.e., multiple “master” versions), each of which has its own local copies. Aligning concepts rather than text documents (i.e., the meanings of metadata terms rather than the documents specifying them) is therefore doubly essential for accurate and machine-automated data aggregation across parallel metadata systems.

**Figure 4:**
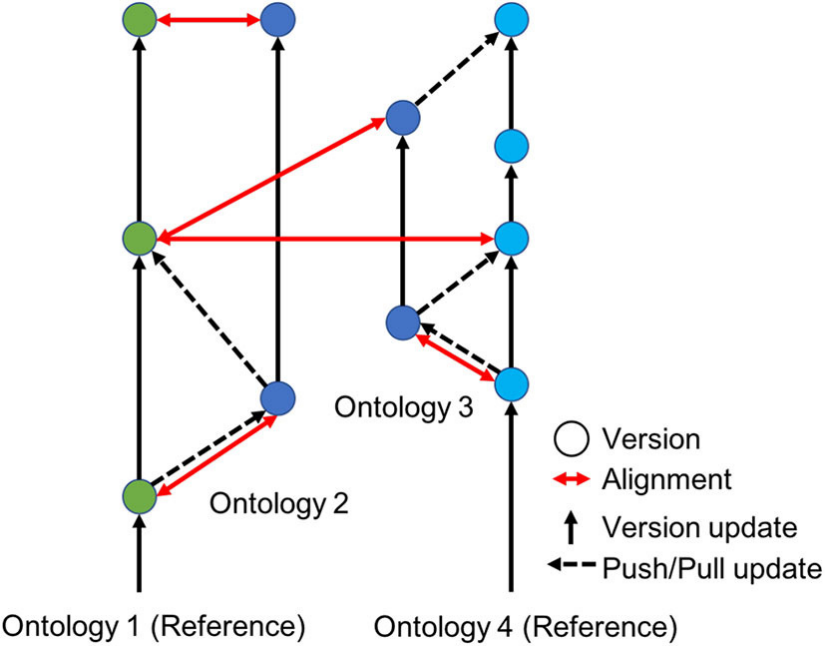
An illustration of semantically-aware decentralized version control for computer ontologies. Ontologies 1 and 2 serve as reference standards (green and light blue dots) shared by different communities of practice, e.g., two taxonomic classifications developed by systematists in North American and Asia for the same perceived group of species. Individual experts or subcommunities can then create local versions of these reference standards (dark blue dots) for research use or to meet local specifications of a government agency or conservation stakeholder. The ability to translate data accurately across local versions and reference standards is provided by ontology alignments (red arrows).

Figure 4 illustrates how our proposed model extends the basic Git model to accommodate semantic alignments and conflict detection among multiple reference standards and thus aggregation of associated datasets. Franz et al. (2016a) have shown how such semantically-aware data translation is possible using logic reasoning based on expert-articulated relationships between metadata categories using Region Connection Calculus-5 (RCC-5). The RCC-5 vocabulary can be understood intuitively as describing five types of relationships between the set of members associated with any pair of concepts. Given two concepts, A and B, RCC-5 lets us say whether A and B congruent, or A is a subset (<) or superset (>), or whether the two regions are overlapping (><) or exclusive of each other B (!). Articulating the relationships between classifications using these relationships enables a computer to reason about how instances of a concept in one classification can be translated into instances of concepts in another classification. A computer is also able to check whether an alignment between two classifications is logically consistent (no contradictory statements about how to translate the data) and comprehensive (every instance of every concept in each classification can be mapped in an unambiguous way onto the other classification).

**Figure 5:**
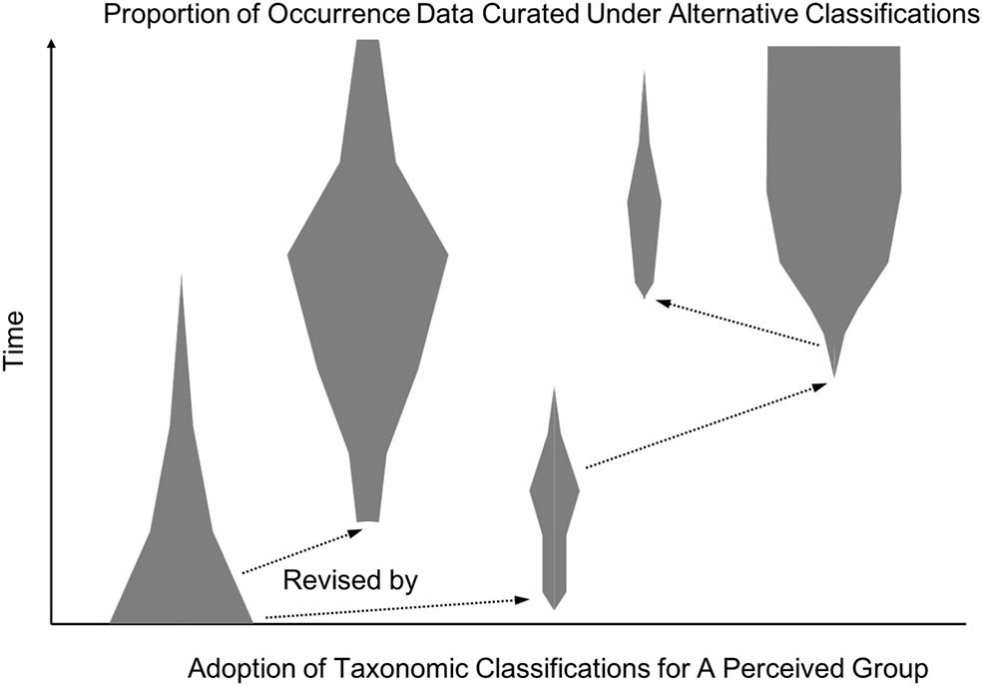
A hypothetical spindle diagram showing the relative usage of taxonomic classifications over time. Each spindle (gray polygons) corresponds to a distinct taxonomic classification for the corresponding (perceived) group, and the spindle's width indicates the relative proportion of occurrence data curated under that classification. The diagram illustrates how multiple classifications may be in heavy use simultaneously, while other proposed revisions do not become widely used but can influence future work (arrows).

Figure 5 then shows how such alignments figure into achieving decentralized data coordination. A community of systematists working on the taxonomy of a group will typically generate multiple proposed classifications over time. Some of these classifications will prove more popular in use for data curation, shown in the figure by the width of the lines. In order to assemble a global data commons, one has to be able to access a comprehensive aggregate of existing data under a single, coherent metadata language. As we discussed with coordinative consensus, this does not entail that all data curation must be done under a single consensus classification. Instead, we need appropriate information about the meanings of the metadata categories within each classification in order to translate across them in an accurate and efficient way. While automated solutions are desirable, high-precision methods for aligning ontologies and detecting conflict remains a challenging research front in computer science (Euzenat and Shvaiko, 2013). It is therefore likely that expert-curated alignments will continue to be necessary for situations where accurate data translation is important for data commons participants.

In sum, lateral alignments across metadata reference standards used by data portals and individual experts can remove the need for a single, globally authoritative consensus classification. Automated reasoning with logical relationships such as in RCC-5 has the potential to lower technical and efficiency barriers to communities developing local consensus classifications customized to their aims and resources. Data portals primarily serving scientists outside systematics, such as conservation biologists or ecologists, may prioritize a more conservative approach to data aggregation based on the most widely used classifications. Alternatively, portals with high levels of crowd-sourced curation may find it valuable to allow contributors to use their preferred classification system out of several options (e.g., iNaturalist, 2019). As Figure 5 suggests, though, alignments between some metadata languages will be more valuable for global data coordination than others. This reflects an important way in which our approach aims to avoid having to pay the labor cost of aligning everything to everything: communities of practice or individual experts are incentivized to connect their preferred datasets and metadata languages to widely shared reference standards, but not at the cost of having their research products overridden or coarse-grained as part of participating in the knowledge commons.

## Developing Socio-Technical Infrastructure to Implement Coordination

**Figure 6:**
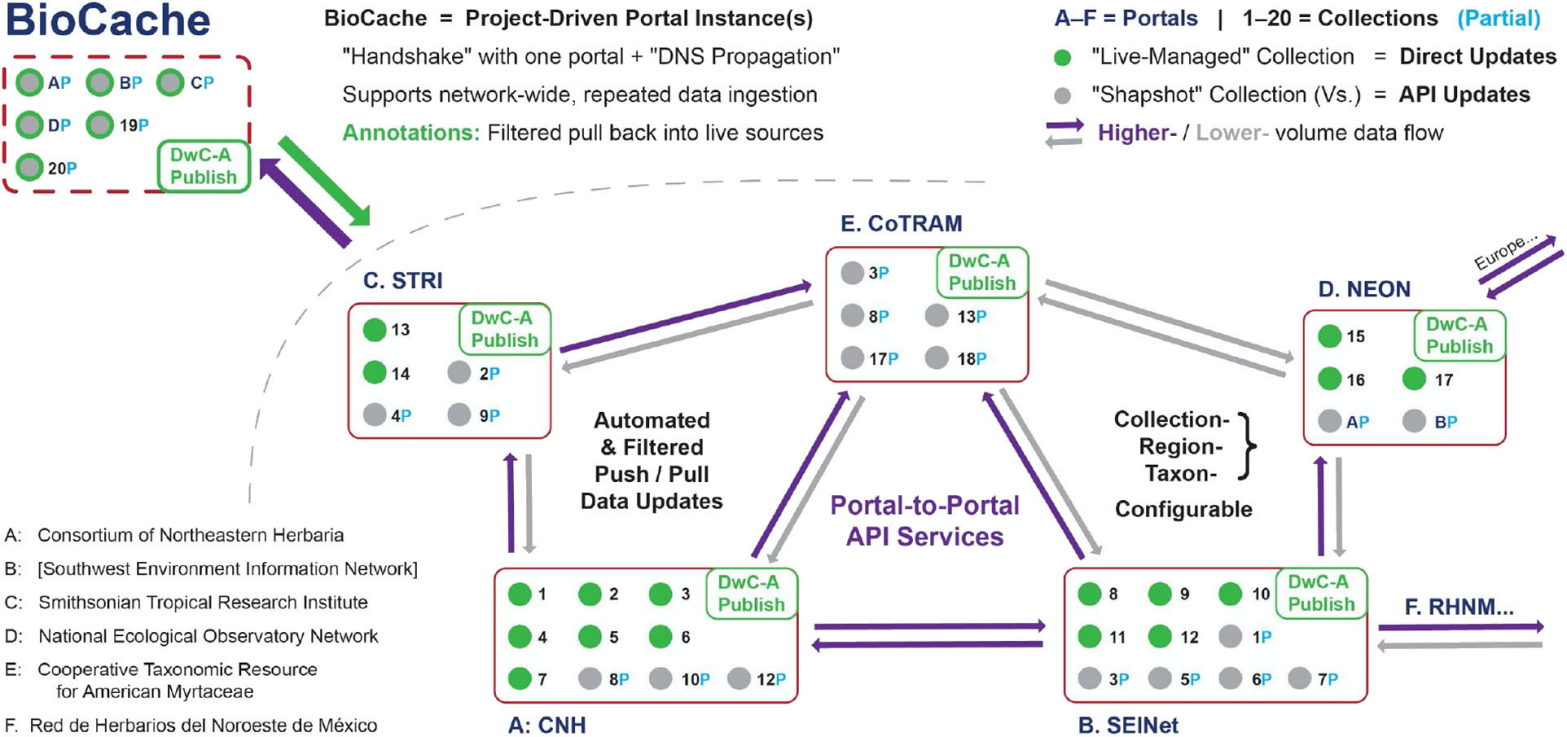
Proposed solution for a decentralized biodiversity data aggregation paradigm, focusing primarily on the interaction between mid-level portal. Low-level entities as in Figure 2 (see explanation of abbreviations and proper names there); however, unlike in Figure 2, the high-level aggregators are no longer relevant. As an essential feature of this proposed design, registration of the BioCache research instance is limited to one network portal; however, propagation of this registration events across the entire portal network is automatically facilitated through mechanisms analogous to Domain Name Server (DNS) propagation. API, Application Programming Interface. See text for additional explanation.

This section uses the Symbiota software platform and portal network to illustrate the social-technical infrastructure we anticipate needing to manage biodiversity data aggregation in a global, decentralized system (Figure 6). We regard this infrastructure as a critical precondition for establishing and nurturing local, high-quality communities of practice for both occurrence records and taxonomic knowledge systems. Our ultimate aim, though, is to illustrate a general solution path that can and should be implemented across many different software and data communities.

Once there is an emerging network of portals whose respective foci and boundaries correspond to active communities of practice, the task of globally coordinating these portals can be achieved through portal-to-portal Application Programming Interface (API) services, which provide socio-technical infrastructure enabling expert knowledge managed within one portal to be shared and coordinated across the portal network. Domain knowledge expertise, for example, could flow out into the extended specimen network with external annotations from other actors then channeled back to the domain expert for review, verification, and acceptance. The function of the API services is thus to “hard-wire” the rights and restrictions for data record and metadata access and editing that maintain the social integrity of each portal community while allowing filtered data annotations (Morris et al., 2013) to flow globally.

A sophisticated cross-portal API encompassing both technical and social components should achieve the following:

- Provide continuous, bi-directional, API-managed data flow across portals and across live-managed vs. snapshot collections.
- Facilitate automated *filtering* of this data flow necessary to represent the social agendas of distributed data and data annotation providers and users.
- Regulate reciprocal updates from portal to portal directly through the API service configuration, including replacements of records with provenance tracking
- Configure and automate portal-to-portal updates such that they are triggered on a regular basis (e.g., nightly).
- Track data modifications based on Globally Unique Identifiers (GUIDs; see Nelson et al., 2018), timestamps, and value changes so that conflicting annotations can be identified and resolved based on a set of rule-based guided decisions.

As an example, data annotations provided by a trusted expert for a particular taxonomic group and region on a snapshot of records in another portal could be allowed through the API configuration to directly become pushed to replace standing data records and annotations in the live-managed collection serving as the reference standard. In other cases, managers for the reference dataset may prefer to review external annotations prior to assigning them “latest viable version” in the live-managed portal. This may include the option to display the new annotations separately in the live-managed collection yet in a linked form, as an alternative view not yet validated by the live-collection manager(s).

Additionally, automatically scheduled network-wide updates would allow all new annotations to be propagated globally (though only laterally; see Figure 5) and in accordance with established rules for propagation and filtering. Such application-to-application synchronization has become standard within the digital world, such as with regular synchronization of data queries, bookmarks, and messages among a person's phones, laptops, and desktop systems.

We illustrate the current status of and progress toward this socio-technical infrastructure using recent projects involving the Symbiota software platform. At present, lateral data flow between mid-level portals using Symbiota software, and hence also between live-managed and snapshot collections represented in these portals, is largely facilitated through batch updates of DwC–A packages that are triggered periodically but also manually, i.e., by an individual (human) with appropriate access rights. For more than a decade, Symbiota has featured a well-developed import module that supports data ingestion from IPT instances, Specify, and other data sources that support DWC-Archive transfer protocols. Similar to the GBIF model, these data pushes typically involve pushing DwC-A data packages from a Live Managed dataset into Snapshot data representations within a remote aggregate Symbiota portal.

These experiences have highlighted the limitations of these methods of transferring data. A significant problem is that data harvesting is limited by the constraints of the data provider, particularly in terms of the content and age of the data. For instance, there is a large US institution that currently publishes their data via an IPT instance that features a single 1.5 GB DwC_archive dataset containing 8.4 million records that span across multiple collection types. This method of publishing not only makes it onerous to harvest and extract taxonomically themed record sets (e.g., lichens, bryophyte, insects), it also hinders the transfer of custom-defined record subsets. Furthermore, it would be ideal if data could be harvested directly from the live dataset, rather than a remote data cache, thus ensuring the most current representation of the data.

These experiences have influenced Symbiota development in several ways. We first summarize two recent steps toward broader implementation among existing portals. We then describe a product in development to provide native support for users to discover, aggregate, curate, and update data records in localized repositories without leaving the network of Symbiota data portals and communities.

With the goal of establishing more flexible, atomized methods of sharing data between Symbiota portals, project developers established a built-in DWC-Archive publishing module that functions similar to the IPT software, though with enhancements that allow more dynamic data sharing. RESTful GET service calls have been established that allow external users to extract custom DwC-Archive data packages from a Live Managed Symbiota dataset based on user-defined filtering criteria (filtered prior to export). For instance, these developments have allowed for the scheduling of automatic data transfers that dynamically extract a fern and fern allies DwC-Archive data package from the University of North Carolina's collection that is managed as a Live Dataset within the SEINet portal and streams the data into a Snapshot representation of the data within the PteridoPortal.

We also have taken steps toward bi-directional data flow with web-service support between Symbiota and an external data management system. Through a collaboration between Symbiota and GeoLocate *(https://www.geo-locate.org/)*, we have developed a set of RESTful API calls and management user interfaces enabling the following workflow:

- Collection managers have the means to define custom subsets of non-georeferenced occurrence records within their collection (e.g., Arizona Poaceae)
- Via RESTful API calls, collection managers can then push record identifiers and verbatim locality description fields for this dataset into a predefined GeoLocate collaborative georeferencing project (GeoLocate CoGe, https://coge.geo-locate.org/)
- As occurrences are georeferenced within the GeoLocate system, coordinates and georeferencing details are streamed back into the source records residing within the Symbiota instance. Coordinate data are pushed into Symbiota via RESTful API calls on a record-by-record basis with full details of the edits maintained within versioning tables.

These projects represent concrete, production-level progress toward a more comprehensive implementation of the features we described earlier in this section.

Looking ahead, we also describe a new product (working title: BioCache), which aims to function as a platform for more narrowly circumscribed communities of practice, e.g., at the scale of individual labs or collaborative projects, while maintaining connectivity with the knowledge commons established by larger and less ephemeral communities such as portals. Scientific analyses of research datasets often take months or years, with data annotations occurring in multiple sites across a project team or collaborative network and creating the need to coordinate data modifications across the network. Research teams, e.g., may also want to ensure that their remotely managed datasets are up-to-date with the distributed network of data providers. At any point in the data package assembly and validation process, mechanisms are needed to pull in additional “upstream” modifications (analogous to a Git pull), which would add new records, append coordinates, and incorporate expert determinations that regularly take place within the collection community.

BioCache therefore aims to provide data discovery tools that allow research teams to harness an API-enabled network of data providers and to define a remote index of occurrence records that will be used to address a particular research project. This data index represents a local versioning of the original, distributed data, and provides a platform where the data can be analyzed, modified, and extended—e.g., error correction, adding of coordinates, occurrence record (re-)identification according to a preferred classification, and annotation of misidentifications—in order to establish a locally robust dataset deemed sufficiently fit to address a specific research interest. In other words, the data index represents a selected “fork” of individual occurrence records analogous to how code forks are defined within GitHub.

Data annotations made in the BioCache instance would be accessible to the original data source via API-driven infrastructure built into the instance's data indexer. This design feature provides the means for the original data provider to selectively pull annotations back into the original, live-managed occurrence record, or at least to define a reference to the extended data product. However, each occurrence record therefore represents its own version fragment that will need to be evaluated, resolved, and merged back into the original data repository on its own terms.

In contrast to a Git code fork, a typical BioCache dataset will consist of occurrences harvested from numerous live-managed portals. Negotiations for a “data fork” to be merged back into the source portals will involve a variety of evaluation and resolution criteria defined by the various agents owning the data. Conversely, research teams will have the means to submit “pull requests” from the remote data fork to all live-managing data providers at once. The latter are offered several options for integrating data annotations back into their live-managed collections and portal. Ideally, occurrence record management tools are enabled with evaluation and resolution mechanisms that can access the research instance's API services to selectively ingest the annotations on the specific terms of the live-managed collection.

## Conclusion

As first-generation projects have matured, the emerging global biodiversity knowledge commons is reaching an important turning point. Globalizing access to occurrence data has generated major successes for science and decision-making, including improved discoverability through aggregator portals and growing adoption of minimum metadata standards such as Darwin Core. However, important limitations have become clear for a top-down strategy emphasizing centralized control over data aggregation and metadata systems. National and international biodiversity aggregators rely on a robust ecosystem of smaller communities of practice focused on particular research themes or collections, but the needs, views, and aims of these communities are at best imperfectly reflected at the global scale. The project of globalizing biodiversity knowledge depends on minimizing harm to these communities' ability to experiment, specialize, and pursue the goals of local stakeholders. Reflecting on current and past efforts in developing the Symbiota platform and portal network, we've presented an innovative approach that can underwrite greater decentralization without sacrificing efficient coordination at the global scale.

Looking forward, decentralized coordination has broader potential to strengthen integration across the biodiversity data life cycle. The future of biodiversity rests, in this regard, finding new ways for computational infrastructure to help scientists and decision-makers resolve complex collective action problems, and we should keep in mind that every technological fix is also a social intervention. Our model emphasizes the development of local communities of practice and data infrastructure aimed at impacting concrete decision-making contexts, rather than a highly standardized, global data product intended to be fit-for-use across most contexts. Scientists are increasingly recognizing the limitations of this latter, loading-dock model for scientific knowledge as a way to influence action (e.g., Cash et al., 2006): simply making all the data available for use in one place is not sufficient to bridge the gap to practical impact, especially if centralization undermines key dimensions of fitness-for-use for the local situations of decision makers. Similarly, research analyzing how data travel effectively across local contexts consistently demonstrate the substantial effort required to design fit-for-use data packaging and reinterpret external datasets for local projects (Gerson, 2008; Leonelli, 2016). Centralized data aggregation can also undermine the long-term value of this information for users, including government stakeholders and conservation planners. Signal distortion due to misaggregation across conflicting taxonomic concepts, for example, has high-stakes implications for species-level assessments of extinction risks.

In this regard, we've argued for the practicality and desirability of providing local data editing rights to communities of practice while maintaining the ability to distribute these updates in a coordinated fashion (i.e., to “trade” in curated datasets; see also Lee, 2017). In a centralized paradigm that forces one-way data flow from sources to users via aggregators, important inefficiencies arise when customization work done to situate data for local use cannot be shared with others. We've also argued for the importance of facilitating how communities of practice transition from informal social networks to formalized governance and infrastructure. A major contributor to the success of Symbiota has been the social component of the application design. Individuals can establish research datasets within their own password-protected user space while maintaining full control over data attribution and management; however, their data are also tightly integrated into a social system which aligns with their research identity. Maintaining full control and ownership of data combined with the ability to exchanged curated content with a community of knowledge experts has been a strong motivating factor to publish data within the Symbiota portal network.

Growing interest in the “extended specimen” program for natural history collections provides an important context for further validation of our approach (Webster, 2017; Lendemer et al., 2020): the next generation of specimen-based research will increasingly augment and integrate already digitized data as well as adding new measurements of physical specimens. This poses new challenges for addressing fragmented occurrence record information across databases and managing novel metadata categories from distributed research projects. BioCache is well-positioned to test the scalability of API-based decentralized versioning with projects producing digitally-native metadata in addition to traditional specimen management.

## Author Contributions

BWS led development of the paper, with primary contributions to theoretical framing of decentralized coordination model (Intro, Sections Growing the Biodiversity Data Commons and A Model for Decentralized But Globally Coordinated Data Aggregation, Conclusion). EEG's primary contributions were to work on the design, description, and implementation of decentralized coordination model (Section Developing Socio-Technical Infrastructure to Implement Coordination). NMF contributed to the overall message/conceptualization of the paper, especially to critique of current model and implementation of alternative for biodiversity data (Sections Reframing the Critique of Globally Centralized Biodiversity Data and Developing Socio-Technical Infrastructure to Implement Coordination).

## Conflict of Interest

The authors declare that the research was conducted in the absence of any commercial or financial relationships that could be construed as a potential conflict of interest.

## Footnotes

^1^Having within- versus outside-portal rights currently coincides with the ability to add or edit records in real-time versus with snapshot updates, but these two aspects could in principle be separated in future implementations.

^2^In principle, though, centralizing occurrence data communities and centralizing taxonomic knowledge could be decoupled and pursued separately.
