# Supplementary material for: Decentralized but Globally Coordinated Biodiversity Data
Source: Front Big Data. 2020 Oct 23;3:519133. doi: 10.3389/fdata.2020.519133 (PMC7931950; doi:10.3389/fdata.2020.519133)
Supplement: Supplementary file 1 [file Table_1.DOCX]

**Supplementary Table**

**Table 1: Design Features for Decentralized but Coordinated Data Model**

| Discoverability | Discoverable data is Web accessible, searchable, and extractable. Domain specific expertise continues to be limited to local systems, so it is important to move data off of individual computers and from behind firewalls on to network enabled systems. Data owners should be enticed with time saving, author-promoting, easy to use applications that are convincingly the best options for their needs. |
| --- | --- |
| Extended attribution | Extend metadata support so that various levels of data subsets can be referenced and attributed to a specific individual or a coordinated group of researchers. Extended attribution will encourage the participation and contribution of domain experts. Need not only to apply attribution to larger datasets, but also to data subset, compiled datasets across collections, taxonomic nodes, identifications, and even individual occurrence edits. Recognizing contributions even at the smallest level encourages buy-in from individual experts. |
| Data control | Dataset owners need to have full control of their data, including the state in which it is published out to the public. However, this does not mean that it needs to be totally immutable and limited to the total control of the owners. External entities should be able to modify or augment data in a controlled and trackable manner (e.g. add coordinates, resolve errors, modify identifications, make comments, map phenology, define extended occurrence attribute, etc). Owners should then be able to easily incorporate or exclude externally generated annotations back into the original source record at their own discretion. |
| Data provenance | Data need to be trackable once a copy of a record leaves the source. This includes maintaining actionable links back to the source with the ability to re-extract a fresh copy of the complete data record. |
| Data conflicts | Conflicts should be allowed and at times even encouraged. Methods that support conflict resolution should be available based on a combination of expert review, group input, and a suite of rating algorithms. Ratings should be adjustable in a manner that allows data consumers to create consensus datasets based on various weighting preferences and selected hierarchical rules. |
| Bidirectional information flow | Applications need API supported bidirectional information flow. Global and midlevel aggregators can add simple annotation interfaces that allow users to edit occurrence records. An edit would consist of edit identifier (guid), occurrence identifier (occurrenceID guid), field name (DwC term), old value, new value, editor name/identifier, and timestamp of edit. |
| Community editing | Any registered user would be allowed to edit records, and edits could be integrated into download interfaces in a manner that would allow data consumers the option of allowing selective edits to take precedence over existing data. This would allow community edits to be selectively applied to abandoned, neglected, or stale occurrence datasets. |
| Edit management interfaces | Collection management applications establish management interfaces that would allow collection administrators to query edits made within aggregators and filter-pull the annotations back into their source records. Ideally, interface would provide displays, visualizations, and rating systems that would collection managers effective evaluation of incoming annotations. |
| Portal-to-portal registry system | A cross-portal handshake registration system that would allow each portal to keep track of other existing portals. When one portal registers with another, registration of the new portal will propagate through the portal network. |
